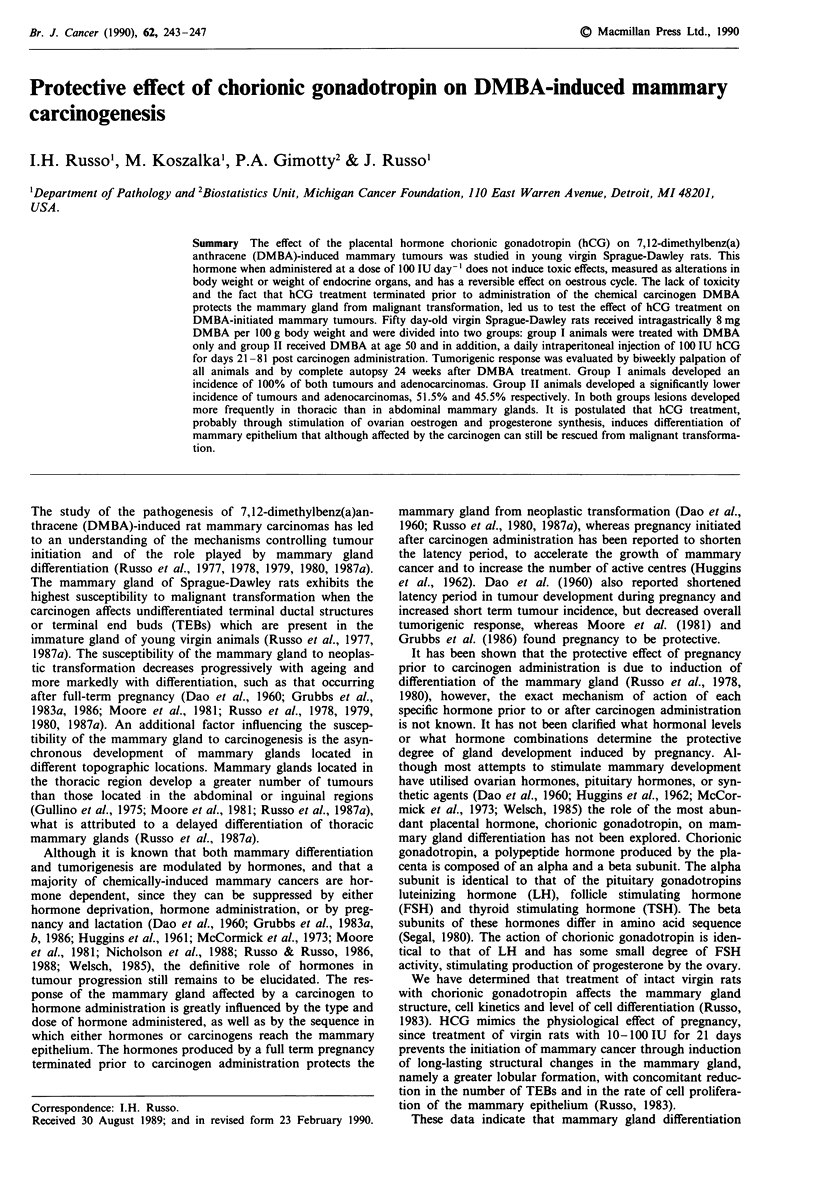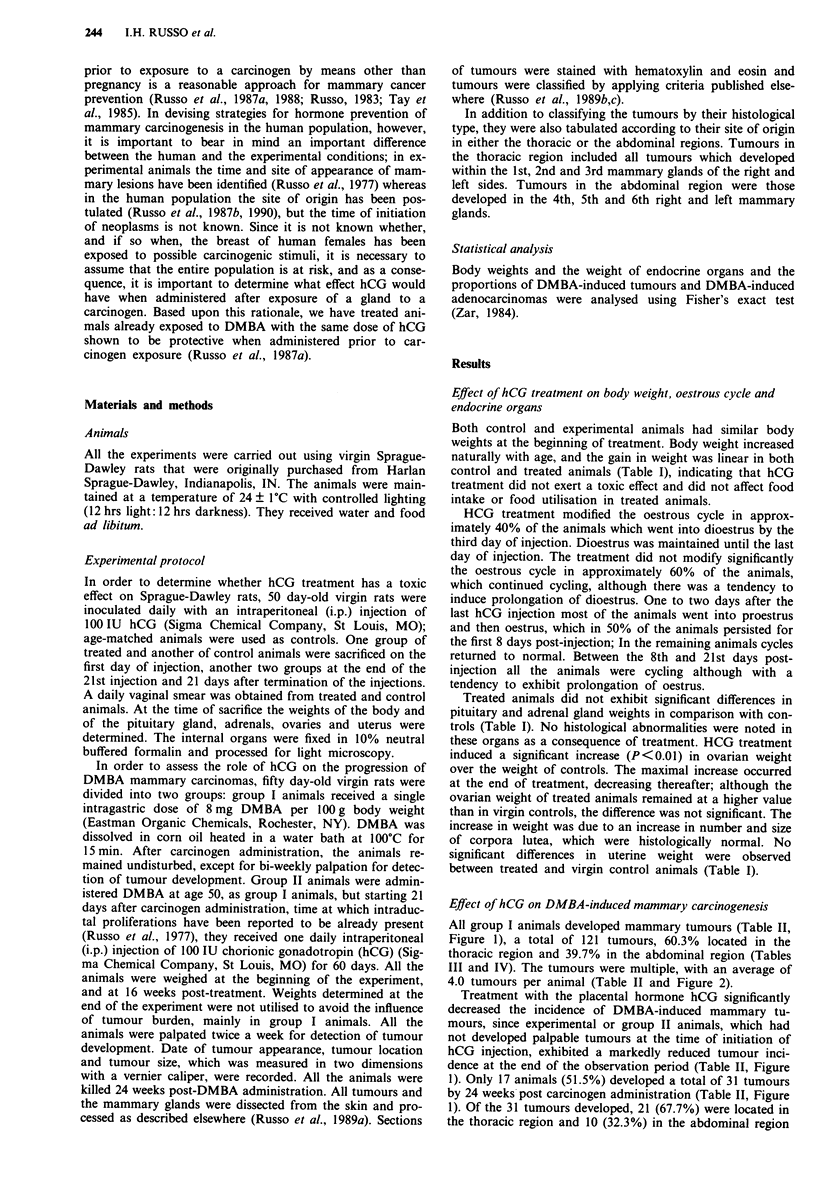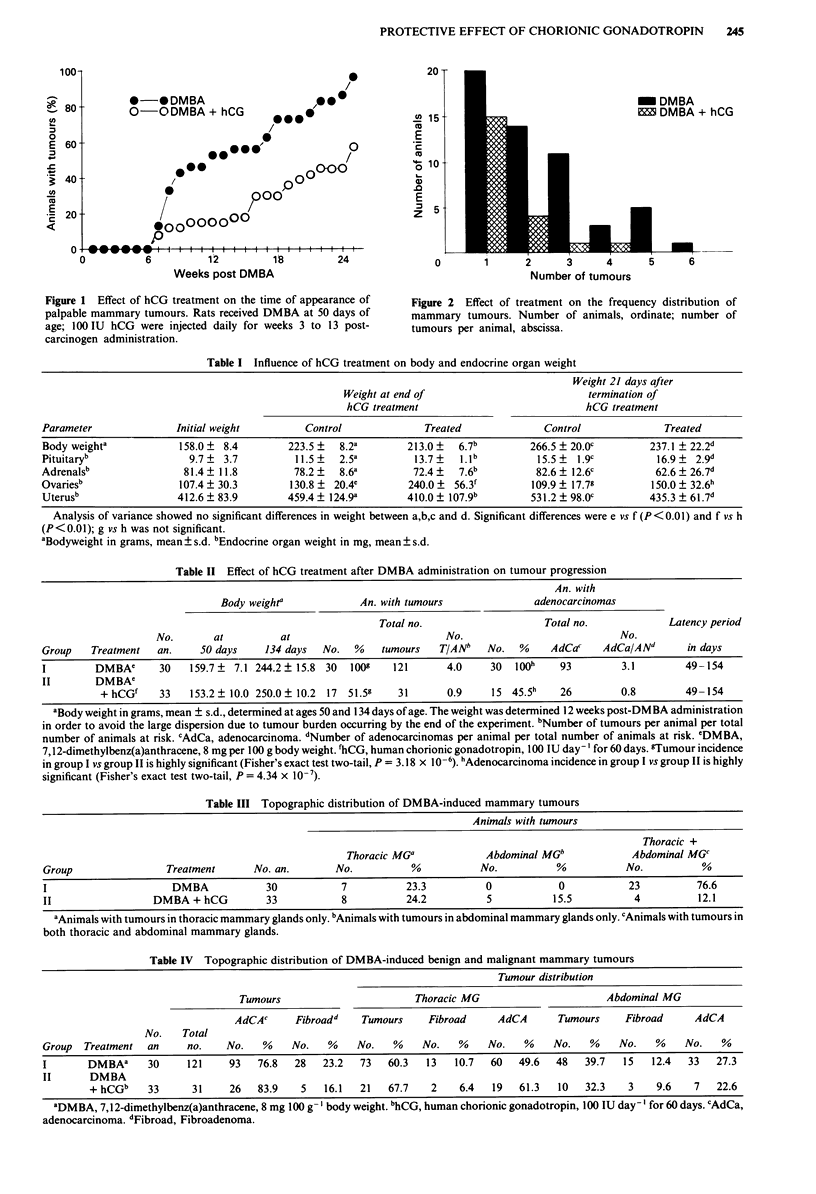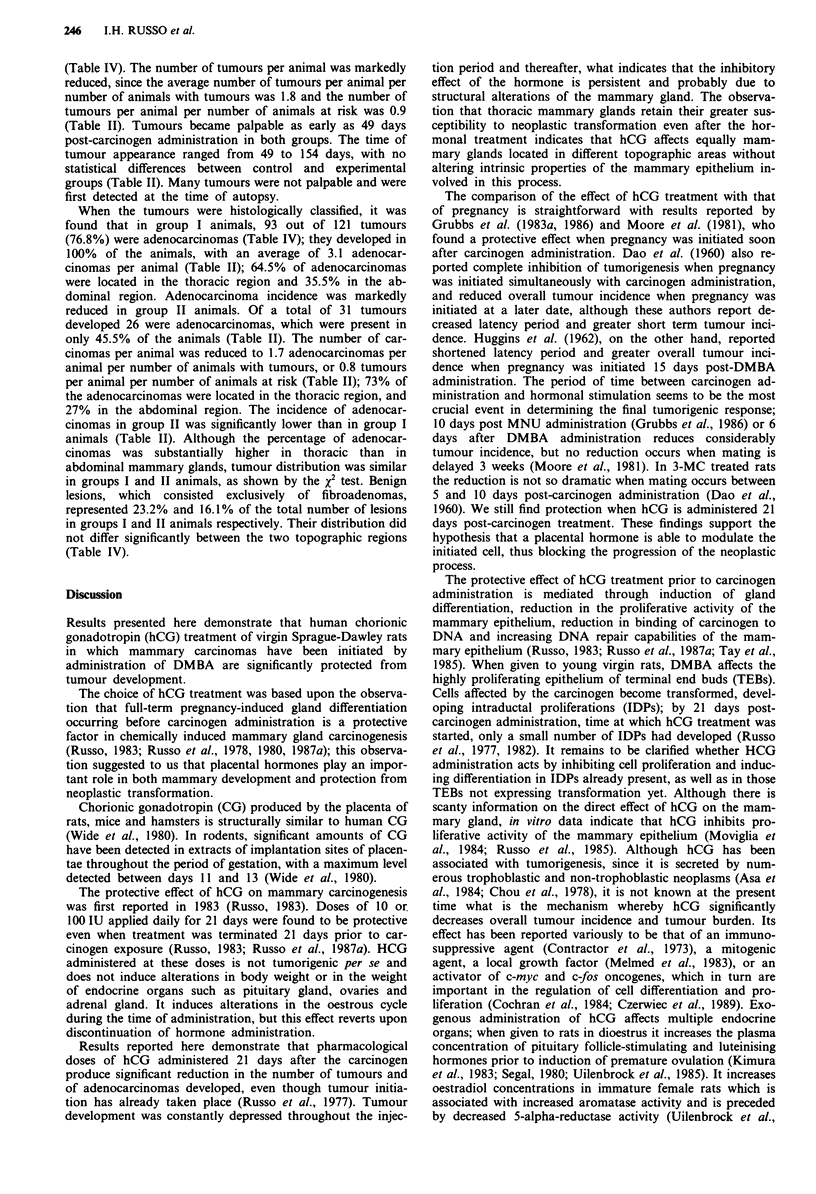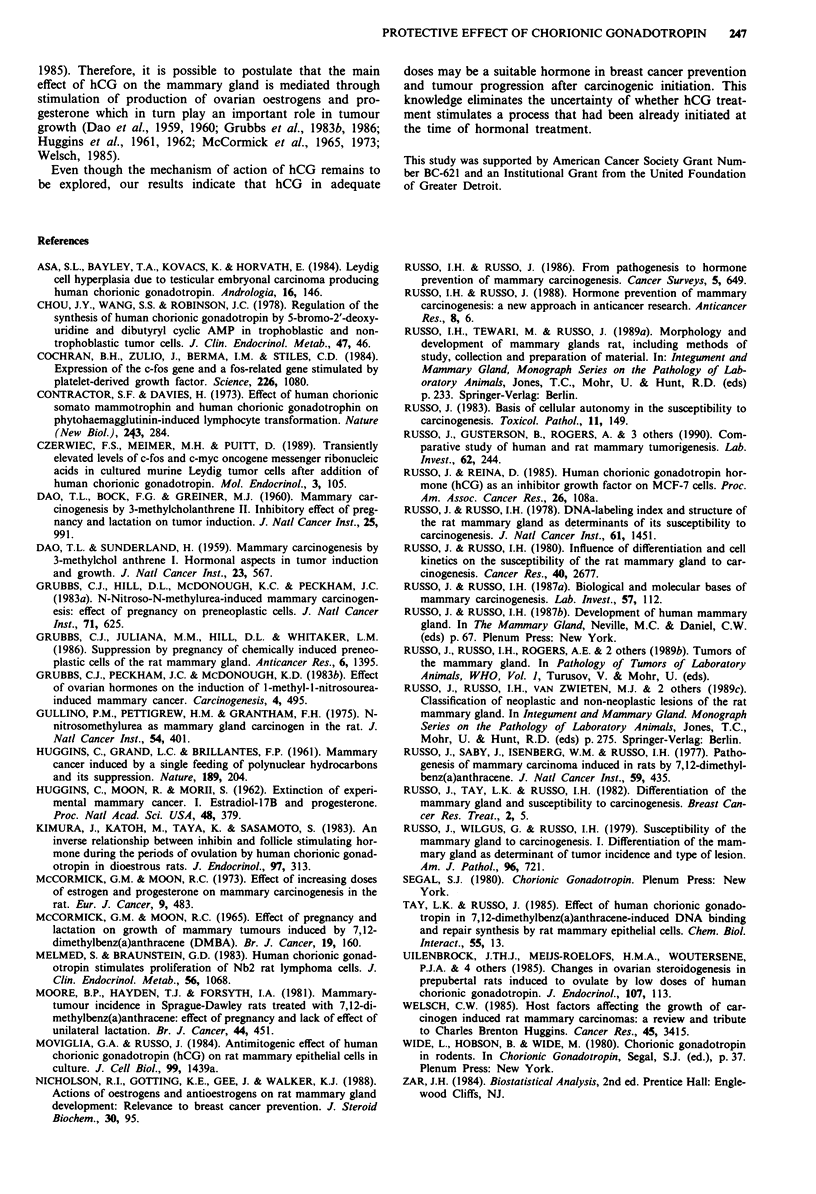# Protective effect of chorionic gonadotropin on DMBA-induced mammary carcinogenesis.

**DOI:** 10.1038/bjc.1990.268

**Published:** 1990-08

**Authors:** I. H. Russo, M. Koszalka, P. A. Gimotty, J. Russo

**Affiliations:** Department of Pathology, Michigan Cancer Foundation, Detroit 48201.

## Abstract

The effect of the placental hormone chorionic gonadotropin (hCG) on 7,12-dimethylbenz(a)anthracene (DMBA)-induced mammary tumours was studied in young virgin Sprague-Dawley rats. This hormone when administered at a dose of 100 IU day-1 does not induce toxic effects, measured as alterations in body weight or weight of endocrine organs, and has a reversible effect on oestrous cycle. The lack of toxicity and the fact that hCG treatment terminated prior to administration of the chemical carcinogen DMBA protects the mammary gland from malignant transformation, led us to test the effect of hCG treatment on DMBA-initiated mammary tumours. Fifty day-old virgin Sprague-Dawley rats received intragastrically 8 mg DMBA per 100 g body weight and were divided into two groups: group I animals were treated with DMBA only and group II received DMBA at age 50 and in addition, a daily intraperitoneal injection of 100 IU hCG for days 21-81 post carcinogen administration. Tumorigenic response was evaluated by biweekly palpation of all animals and by complete autopsy 24 weeks after DMBA treatment. Group I animals developed an incidence of 100% of both tumours and adenocarcinomas. Group II animals developed a significantly lower incidence of tumours and adenocarcinomas, 51.5% and 45.5% respectively. In both groups lesions developed more frequently in thoracic than in abdominal mammary glands. It is postulated that hCG treatment, probably through stimulation of ovarian oestrogen and progesterone synthesis, induces differentiation of mammary epithelium that although affected by the carcinogen can still be rescued from malignant transformation.


*Br. J. Cancer* (1990), **62**, 243–247 © Macmillan Press Ltd., 1990

### Protective effect of chorionic gonadotropin on DMBA-induced mammary carcinogenesis

I.H. Russo[1], M. Koszalka[1], P.A. Gimotty[2] & J. Russo[1]

[1]*Department of Pathology and* [2]*Biostatistics Unit, Michigan Cancer Foundation, 110 East Warren Avenue, Detroit, MI 48201, USA.*

**Summary** The effect of the placental hormone chorionic gonadotropin (hCG) on 7,12-dimethylbenz(a) anthracene (DMBA)-induced mammary tumours was studied in young virgin Sprague-Dawley rats. This hormone when administered at a dose of 100 IU day$^{-1}$ does not induce toxic effects, measured as alterations in body weight or weight of endocrine organs, and has a reversible effect on oestrous cycle. The lack of toxicity and the fact that hCG treatment terminated prior to administration of the chemical carcinogen DMBA protects the mammary gland from malignant transformation, led us to test the effect of hCG treatment on DMBA-initiated mammary tumours. Fifty day-old virgin Sprague-Dawley rats received intragastrically 8 mg DMBA per 100 g body weight and were divided into two groups: group I animals were treated with DMBA only and group II received DMBA at age 50 and in addition, a daily intraperitoneal injection of 100 IU hCG for days 21–81 post carcinogen administration. Tumorigenic response was evaluated by biweekly palpation of all animals and by complete autopsy 24 weeks after DMBA treatment. Group I animals developed an incidence of 100% of both tumours and adenocarcinomas. Group II animals developed a significantly lower incidence of tumours and adenocarcinomas, 51.5% and 45.5% respectively. In both groups lesions developed more frequently in thoracic than in abdominal mammary glands. It is postulated that hCG treatment, probably through stimulation of ovarian oestrogen and progesterone synthesis, induces differentiation of mammary epithelium that although affected by the carcinogen can still be rescued from malignant transformation.

The study of the pathogenesis of 7,12-dimethylbenz(a)anthracene (DMBA)-induced rat mammary carcinomas has led to an understanding of the mechanisms controlling tumour initiation and of the role played by mammary gland differentiation (Russo *et al.*, 1977, 1978, 1979, 1980, 1987*a*). The mammary gland of Sprague-Dawley rats exhibits the highest susceptibility to malignant transformation when the carcinogen affects undifferentiated terminal ductal structures or terminal end buds (TEBs) which are present in the immature gland of young virgin animals (Russo *et al.*, 1977, 1987*a*). The susceptibility of the mammary gland to neoplastic transformation decreases progressively with ageing and more markedly with differentiation, such as that occurring after full-term pregnancy (Dao *et al.*, 1960; Grubbs *et al.*, 1983*a*, 1986; Moore *et al.*, 1981; Russo *et al.*, 1978, 1979, 1980, 1987*a*). An additional factor influencing the susceptibility of the mammary gland to carcinogenesis is the asynchronous development of mammary glands located in different topographic locations. Mammary glands located in the thoracic region develop a greater number of tumours than those located in the abdominal or inguinal regions (Gullino *et al.*, 1975; Moore *et al.*, 1981; Russo *et al.*, 1987*a*), what is attributed to a delayed differentiation of thoracic mammary glands (Russo *et al.*, 1987*a*).

Although it is known that both mammary differentiation and tumorigenesis are modulated by hormones, and that a majority of chemically-induced mammary cancers are hormone dependent, since they can be suppressed by either hormone deprivation, hormone administration, or by pregnancy and lactation (Dao *et al.*, 1960; Grubbs *et al.*, 1983*a*, *b*, 1986; Huggins *et al.*, 1961; McCormick *et al.*, 1973; Moore *et al.*, 1981; Nicholson *et al.*, 1988; Russo & Russo, 1986, 1988; Welsch, 1985), the definitive role of hormones in tumour progression still remains to be elucidated. The response of the mammary gland affected by a carcinogen to hormone administration is greatly influenced by the type and dose of hormone administered, as well as by the sequence in which either hormones or carcinogens reach the mammary epithelium. The hormones produced by a full term pregnancy terminated prior to carcinogen administration protects the mammary gland from neoplastic transformation (Dao *et al.*, 1960; Russo *et al.*, 1980, 1987*a*), whereas pregnancy initiated after carcinogen administration has been reported to shorten the latency period, to accelerate the growth of mammary cancer and to increase the number of active centres (Huggins *et al.*, 1962). Dao *et al.* (1960) also reported shortened latency period in tumour development during pregnancy and increased short term tumour incidence, but decreased overall tumorigenic response, whereas Moore *et al.* (1981) and Grubbs *et al.* (1986) found pregnancy to be protective.

It has been shown that the protective effect of pregnancy prior to carcinogen administration is due to induction of differentiation of the mammary gland (Russo *et al.*, 1978, 1980), however, the exact mechanism of action of each specific hormone prior to or after carcinogen administration is not known. It has not been clarified what hormonal levels or what hormone combinations determine the protective degree of gland development induced by pregnancy. Although most attempts to stimulate mammary development have utilised ovarian hormones, pituitary hormones, or synthetic agents (Dao *et al.*, 1960; Huggins *et al.*, 1962; McCormick *et al.*, 1973; Welsch, 1985) the role of the most abundant placental hormone, chorionic gonadotropin, on mammary gland differentiation has not been explored. Chorionic gonadotropin, a polypeptide hormone produced by the placenta is composed of an alpha and a beta subunit. The alpha subunit is identical to that of the pituitary gonadotropins luteinizing hormone (LH), follicle stimulating hormone (FSH) and thyroid stimulating hormone (TSH). The beta subunits of these hormones differ in amino acid sequence (Segal, 1980). The action of chorionic gonadotropin is identical to that of LH and has some small degree of FSH activity, stimulating production of progesterone by the ovary.

We have determined that treatment of intact virgin rats with chorionic gonadotropin affects the mammary gland structure, cell kinetics and level of cell differentiation (Russo, 1983). HCG mimics the physiological effect of pregnancy, since treatment of virgin rats with 10–100 IU for 21 days prevents the initiation of mammary cancer through induction of long-lasting structural changes in the mammary gland, namely a greater lobular formation, with concomitant reduction in the number of TEBs and in the rate of cell proliferation of the mammary epithelium (Russo, 1983).

These data indicate that mammary gland differentiation

Correspondence: I.H. Russo.
Received 30 August 1989; and in revised form 23 February 1990.

prior to exposure to a carcinogen by means other than pregnancy is a reasonable approach for mammary cancer prevention (Russo *et al.*, 1987*a*, 1988; Russo, 1983; Tay *et al.*, 1985). In devising strategies for hormone prevention of mammary carcinogenesis in the human population, however, it is important to bear in mind an important difference between the human and the experimental conditions; in experimental animals the time and site of appearance of mammary lesions have been identified (Russo *et al.*, 1977) whereas in the human population the site of origin has been postulated (Russo *et al.*, 1987*b*, 1990), but the time of initiation of neoplasms is not known. Since it is not known whether, and if so when, the breast of human females has been exposed to possible carcinogenic stimuli, it is necessary to assume that the entire population is at risk, and as a consequence, it is important to determine what effect hCG would have when administered after exposure of a gland to a carcinogen. Based upon this rationale, we have treated animals already exposed to DMBA with the same dose of hCG shown to be protective when administered prior to carcinogen exposure (Russo *et al.*, 1987*a*).

### Materials and methods

### *Animals*

All the experiments were carried out using virgin Sprague-Dawley rats that were originally purchased from Harlan Sprague-Dawley, Indianapolis, IN. The animals were maintained at a temperature of 24 ± 1°C with controlled lighting (12 hrs light: 12 hrs darkness). They received water and food *ad libitum*.

### *Experimental protocol*

In order to determine whether hCG treatment has a toxic effect on Sprague-Dawley rats, 50 day-old virgin rats were inoculated daily with an intraperitoneal (i.p.) injection of 100 IU hCG (Sigma Chemical Company, St Louis, MO); age-matched animals were used as controls. One group of treated and another of control animals were sacrificed on the first day of injection, another two groups at the end of the 21st injection and 21 days after termination of the injections. A daily vaginal smear was obtained from treated and control animals. At the time of sacrifice the weights of the body and of the pituitary gland, adrenals, ovaries and uterus were determined. The internal organs were fixed in 10% neutral buffered formalin and processed for light microscopy.

In order to assess the role of hCG on the progression of DMBA mammary carcinomas, fifty day-old virgin rats were divided into two groups: group I animals received a single intragastric dose of 8 mg DMBA per 100 g body weight (Eastman Organic Chemicals, Rochester, NY). DMBA was dissolved in corn oil heated in a water bath at 100°C for 15 min. After carcinogen administration, the animals remained undisturbed, except for bi-weekly palpation for detection of tumour development. Group II animals were administered DMBA at age 50, as group I animals, but starting 21 days after carcinogen administration, time at which intraductal proliferations have been reported to be already present (Russo *et al.*, 1977), they received one daily intraperitoneal (i.p.) injection of 100 IU chorionic gonadotropin (hCG) (Sigma Chemical Company, St Louis, MO) for 60 days. All the animals were weighed at the beginning of the experiment, and at 16 weeks post-treatment. Weights determined at the end of the experiment were not utilised to avoid the influence of tumour burden, mainly in group I animals. All the animals were palpated twice a week for detection of tumour development. Date of tumour appearance, tumour location and tumour size, which was measured in two dimensions with a vernier caliper, were recorded. All the animals were killed 24 weeks post-DMBA administration. All tumours and the mammary glands were dissected from the skin and processed as described elsewhere (Russo *et al.*, 1989*a*). Sections of tumours were stained with hematoxylin and eosin and tumours were classified by applying criteria published elsewhere (Russo *et al.*, 1989*b*,*c*).

In addition to classifying the tumours by their histological type, they were also tabulated according to their site of origin in either the thoracic or the abdominal regions. Tumours in the thoracic region included all tumours which developed within the 1st, 2nd and 3rd mammary glands of the right and left sides. Tumours in the abdominal region were those developed in the 4th, 5th and 6th right and left mammary glands.

### *Statistical analysis*

Body weights and the weight of endocrine organs and the proportions of DMBA-induced tumours and DMBA-induced adenocarcinomas were analysed using Fisher's exact test (Zar, 1984).

### Results

### *Effect of hCG treatment on body weight, oestrous cycle and endocrine organs*

Both control and experimental animals had similar body weights at the beginning of treatment. Body weight increased naturally with age, and the gain in weight was linear in both control and treated animals (Table I), indicating that hCG treatment did not exert a toxic effect and did not affect food intake or food utilisation in treated animals.

HCG treatment modified the oestrous cycle in approximately 40% of the animals which went into dioestrus by the third day of injection. Dioestrus was maintained until the last day of injection. The treatment did not modify significantly the oestrous cycle in approximately 60% of the animals, which continued cycling, although there was a tendency to induce prolongation of dioestrus. One to two days after the last hCG injection most of the animals went into proestrus and then oestrus, which in 50% of the animals persisted for the first 8 days post-injection; In the remaining animals cycles returned to normal. Between the 8th and 21st days post-injection all the animals were cycling although with a tendency to exhibit prolongation of oestrus.

Treated animals did not exhibit significant differences in pituitary and adrenal gland weights in comparison with controls (Table I). No histological abnormalities were noted in these organs as a consequence of treatment. HCG treatment induced a significant increase ($P < 0.01$) in ovarian weight over the weight of controls. The maximal increase occurred at the end of treatment, decreasing thereafter; although the ovarian weight of treated animals remained at a higher value than in virgin controls, the difference was not significant. The increase in weight was due to an increase in number and size of corpora lutea, which were histologically normal. No significant differences in uterine weight were observed between treated and virgin control animals (Table I).

### *Effect of hCG on DMBA-induced mammary carcinogenesis*

All group I animals developed mammary tumours (Table II, Figure 1), a total of 121 tumours, 60.3% located in the thoracic region and 39.7% in the abdominal region (Tables III and IV). The tumours were multiple, with an average of 4.0 tumours per animal (Table II and Figure 2).

Treatment with the placental hormone hCG significantly decreased the incidence of DMBA-induced mammary tumours, since experimental or group II animals, which had not developed palpable tumours at the time of initiation of hCG injection, exhibited a markedly reduced tumour incidence at the end of the observation period (Table II, Figure 1). Only 17 animals (51.5%) developed a total of 31 tumours by 24 weeks post carcinogen administration (Table II, Figure 1). Of the 31 tumours developed, 21 (67.7%) were located in the thoracic region and 10 (32.3%) in the abdominal region

**Figure 1** Effect of hCG treatment on the time of appearance of palpable mammary tumours. Rats received DMBA at 50 days of age; 100 IU hCG were injected daily for weeks 3 to 13 post-carcinogen administration.

**Figure 2** Effect of treatment on the frequency distribution of mammary tumours. Number of animals, ordinate; number of tumours per animal, abscissa.

**Table I** Influence of hCG treatment on body and endocrine organ weight

| | | *Weight at end of hCG treatment* | | *Weight 21 days after termination of hCG treatment* | |
|---|---|---|---|---|---|
| *Parameter* | *Initial weight* | *Control* | *Treated* | *Control* | *Treated* |
| Body weight[a] | 158.0 ± 8.4 | 223.5 ± 8.2[a] | 213.0 ± 6.7[b] | 266.5 ± 20.0[c] | 237.1 ± 22.2[d] |
| Pituitary[b] | 9.7 ± 3.7 | 11.5 ± 2.5[a] | 13.7 ± 1.1[b] | 15.5 ± 1.9[c] | 16.9 ± 2.9[d] |
| Adrenals[b] | 81.4 ± 11.8 | 78.2 ± 8.6[a] | 72.4 ± 7.6[b] | 82.6 ± 12.6[c] | 62.6 ± 26.7[d] |
| Ovaries[b] | 107.4 ± 30.3 | 130.8 ± 20.4[e] | 240.0 ± 56.3[f] | 109.9 ± 17.7[g] | 150.0 ± 32.6[h] |
| Uterus[b] | 412.6 ± 83.9 | 459.4 ± 124.9[a] | 410.0 ± 107.9[b] | 531.2 ± 98.0[c] | 435.3 ± 61.7[d] |

Analysis of variance showed no significant differences in weight between a,b,c and d. Significant differences were e *vs* f ($P<0.01$) and f *vs* h ($P<0.01$); g *vs* h was not significant.
[a]Bodyweight in grams, mean ± s.d. [b]Endocrine organ weight in mg, mean ± s.d.

**Table II** Effect of hCG treatment after DMBA administration on tumour progression

| | | | *Body weight[a]* | | *An. with tumours* | | | | *An. with adenocarcinomas* | | | | |
|---|---|---|---|---|---|---|---|---|---|---|---|---|---|
| *Group* | *Treatment* | *No. an.* | *at 50 days* | *at 134 days* | *No.* | *%* | *Total no. tumours* | *No. T/AN[b]* | *No.* | *%* | *Total no. AdCa[c]* | *No. AdCa/AN[d]* | *Latency period in days* |
| I | DMBA[e] | 30 | 159.7 ± 7.1 | 244.2 ± 15.8 | 30 | 100[g] | 121 | 4.0 | 30 | 100[h] | 93 | 3.1 | 49–154 |
| II | DMBA[e] + hCG[f] | 33 | 153.2 ± 10.0 | 250.0 ± 10.2 | 17 | 51.5[g] | 31 | 0.9 | 15 | 45.5[h] | 26 | 0.8 | 49–154 |

[a]Body weight in grams, mean ± s.d., determined at ages 50 and 134 days of age. The weight was determined 12 weeks post-DMBA administration in order to avoid the large dispersion due to tumour burden occurring by the end of the experiment. [b]Number of tumours per animal per total number of animals at risk. [c]AdCa, adenocarcinoma. [d]Number of adenocarcinomas per animal per total number of animals at risk. [e]DMBA, 7,12-dimethylbenz(a)anthracene, 8 mg per 100 g body weight. [f]hCG, human chorionic gonadotropin, 100 IU $day^{-1}$ for 60 days. [g]Tumour incidence in group I *vs* group II is highly significant (Fisher's exact test two-tail, $P = 3.18 \times 10^{-6}$). [h]Adenocarcinoma incidence in group I *vs* group II is highly significant (Fisher's exact test two-tail, $P = 4.34 \times 10^{-7}$).

**Table III** Topographic distribution of DMBA-induced mammary tumours

| | | | *Animals with tumours* | | | | | |
|---|---|---|---|---|---|---|---|---|
| | | | *Thoracic MG[a]* | | *Abdominal MG[b]* | | *Thoracic + Abdominal MG[c]* | |
| *Group* | *Treatment* | *No. an.* | *No.* | *%* | *No.* | *%* | *No.* | *%* |
| I | DMBA | 30 | 7 | 23.3 | 0 | 0 | 23 | 76.6 |
| II | DMBA + hCG | 33 | 8 | 24.2 | 5 | 15.5 | 4 | 12.1 |

[a]Animals with tumours in thoracic mammary glands only. [b]Animals with tumours in abdominal mammary glands only. [c]Animals with tumours in both thoracic and abdominal mammary glands.

**Table IV** Topographic distribution of DMBA-induced benign and malignant mammary tumours

| | | | *Tumours* | | | | | *Tumour distribution* | | | | | | | | | | | | |
|---|---|---|---|---|---|---|---|---|---|---|---|---|---|---|---|---|---|---|---|---|
| | | | | | | | | *Thoracic MG* | | | | | | *Abdominal MG* | | | | | | |
| | | | | *AdCA[c]* | | *Fibroad[d]* | | *Tumours* | | *Fibroad* | | *AdCA* | | *Tumours* | | *Fibroad* | | *AdCA* | | |
| *Group* | *Treatment* | *No. an* | *Total no.* | *No.* | *%* | *No.* | *%* | *No.* | *%* | *No.* | *%* | *No.* | *%* | *No.* | *%* | *No.* | *%* | *No.* | *%* | |
| I | DMBA[a] | 30 | 121 | 93 | 76.8 | 28 | 23.2 | 73 | 60.3 | 13 | 10.7 | 60 | 49.6 | 48 | 39.7 | 15 | 12.4 | 33 | 27.3 | |
| II | DMBA + hCG[b] | 33 | 31 | 26 | 83.9 | 5 | 16.1 | 21 | 67.7 | 2 | 6.4 | 19 | 61.3 | 10 | 32.3 | 3 | 9.6 | 7 | 22.6 | |

[a]DMBA, 7,12-dimethylbenz(a)anthracene, 8 mg 100 $g^{-1}$ body weight. [b]hCG, human chorionic gonadotropin, 100 IU $day^{-1}$ for 60 days. [c]AdCa, adenocarcinoma. [d]Fibroad, Fibroadenoma.

(Table IV). The number of tumours per animal was markedly reduced, since the average number of tumours per animal per number of animals with tumours was 1.8 and the number of tumours per animal per number of animals at risk was 0.9 (Table II). Tumours became palpable as early as 49 days post-carcinogen administration in both groups. The time of tumour appearance ranged from 49 to 154 days, with no statistical differences between control and experimental groups (Table II). Many tumours were not palpable and were first detected at the time of autopsy.

When the tumours were histologically classified, it was found that in group I animals, 93 out of 121 tumours (76.8%) were adenocarcinomas (Table IV); they developed in 100% of the animals, with an average of 3.1 adenocarcinomas per animal (Table II); 64.5% of adenocarcinomas were located in the thoracic region and 35.5% in the abdominal region. Adenocarcinoma incidence was markedly reduced in group II animals. Of a total of 31 tumours developed 26 were adenocarcinomas, which were present in only 45.5% of the animals (Table II). The number of carcinomas per animal was reduced to 1.7 adenocarcinomas per animal per number of animals with tumours, or 0.8 tumours per animal per number of animals at risk (Table II); 73% of the adenocarcinomas were located in the thoracic region, and 27% in the abdominal region. The incidence of adenocarcinomas in group II was significantly lower than in group I animals (Table II). Although the percentage of adenocarcinomas was substantially higher in thoracic than in abdominal mammary glands, tumour distribution was similar in groups I and II animals, as shown by the $\chi^2$ test. Benign lesions, which consisted exclusively of fibroadenomas, represented 23.2% and 16.1% of the total number of lesions in groups I and II animals respectively. Their distribution did not differ significantly between the two topographic regions (Table IV).

### Discussion

Results presented here demonstrate that human chorionic gonadotropin (hCG) treatment of virgin Sprague-Dawley rats in which mammary carcinomas have been initiated by administration of DMBA are significantly protected from tumour development.

The choice of hCG treatment was based upon the observation that full-term pregnancy-induced gland differentiation occurring before carcinogen administration is a protective factor in chemically induced mammary gland carcinogenesis (Russo, 1983; Russo *et al.*, 1978, 1980, 1987*a*); this observation suggested to us that placental hormones play an important role in both mammary development and protection from neoplastic transformation.

Chorionic gonadotropin (CG) produced by the placenta of rats, mice and hamsters is structurally similar to human CG (Wide *et al.*, 1980). In rodents, significant amounts of CG have been detected in extracts of implantation sites of placentae throughout the period of gestation, with a maximum level detected between days 11 and 13 (Wide *et al.*, 1980).

The protective effect of hCG on mammary carcinogenesis was first reported in 1983 (Russo, 1983). Doses of 10 or 100 IU applied daily for 21 days were found to be protective even when treatment was terminated 21 days prior to carcinogen exposure (Russo, 1983; Russo *et al.*, 1987*a*). HCG administered at these doses is not tumorigenic *per se* and does not induce alterations in body weight or in the weight of endocrine organs such as pituitary gland, ovaries and adrenal gland. It induces alterations in the oestrous cycle during the time of administration, but this effect reverts upon discontinuation of hormone administration.

Results reported here demonstrate that pharmacological doses of hCG administered 21 days after the carcinogen produce significant reduction in the number of tumours and of adenocarcinomas developed, even though tumour initiation has already taken place (Russo *et al.*, 1977). Tumour development was constantly depressed throughout the injection period and thereafter, what indicates that the inhibitory effect of the hormone is persistent and probably due to structural alterations of the mammary gland. The observation that thoracic mammary glands retain their greater susceptibility to neoplastic transformation even after the hormonal treatment indicates that hCG affects equally mammary glands located in different topographic areas without altering intrinsic properties of the mammary epithelium involved in this process.

The comparison of the effect of hCG treatment with that of pregnancy is straightforward with results reported by Grubbs *et al.* (1983*a*, 1986) and Moore *et al.* (1981), who found a protective effect when pregnancy was initiated soon after carcinogen administration. Dao *et al.* (1960) also reported complete inhibition of tumorigenesis when pregnancy was initiated simultaneously with carcinogen administration, and reduced overall tumour incidence when pregnancy was initiated at a later date, although these authors report decreased latency period and greater short term tumour incidence. Huggins *et al.* (1962), on the other hand, reported shortened latency period and greater overall tumour incidence when pregnancy was initiated 15 days post-DMBA administration. The period of time between carcinogen administration and hormonal stimulation seems to be the most crucial event in determining the final tumorigenic response; 10 days post MNU administration (Grubbs *et al.*, 1986) or 6 days after DMBA administration reduces considerably tumour incidence, but no reduction occurs when mating is delayed 3 weeks (Moore *et al.*, 1981). In 3-MC treated rats the reduction is not so dramatic when mating occurs between 5 and 10 days post-carcinogen administration (Dao *et al.*, 1960). We still find protection when hCG is administered 21 days post-carcinogen treatment. These findings support the hypothesis that a placental hormone is able to modulate the initiated cell, thus blocking the progression of the neoplastic process.

The protective effect of hCG treatment prior to carcinogen administration is mediated through induction of gland differentiation, reduction in the proliferative activity of the mammary epithelium, reduction in binding of carcinogen to DNA and increasing DNA repair capabilities of the mammary epithelium (Russo, 1983; Russo *et al.*, 1987*a*; Tay *et al.*, 1985). When given to young virgin rats, DMBA affects the highly proliferating epithelium of terminal end buds (TEBs). Cells affected by the carcinogen become transformed, developing intraductal proliferations (IDPs); by 21 days post-carcinogen administration, time at which hCG treatment was started, only a small number of IDPs had developed (Russo *et al.*, 1977, 1982). It remains to be clarified whether HCG administration acts by inhibiting cell proliferation and inducing differentiation in IDPs already present, as well as in those TEBs not expressing transformation yet. Although there is scanty information on the direct effect of hCG on the mammary gland, *in vitro* data indicate that hCG inhibits proliferative activity of the mammary epithelium (Moviglia *et al.*, 1984; Russo *et al.*, 1985). Although hCG has been associated with tumorigenesis, since it is secreted by numerous trophoblastic and non-trophoblastic neoplasms (Asa *et al.*, 1984; Chou *et al.*, 1978), it is not known at the present time what is the mechanism whereby hCG significantly decreases overall tumour incidence and tumour burden. Its effect has been reported variously to be that of an immunosuppressive agent (Contractor *et al.*, 1973), a mitogenic agent, a local growth factor (Melmed *et al.*, 1983), or an activator of c-*myc* and c-*fos* oncogenes, which in turn are important in the regulation of cell differentiation and proliferation (Cochran *et al.*, 1984; Czerwiec *et al.*, 1989). Exogenous administration of hCG affects multiple endocrine organs; when given to rats in dioestrus it increases the plasma concentration of pituitary follicle-stimulating and luteinising hormones prior to induction of premature ovulation (Kimura *et al.*, 1983; Segal, 1980; Uilenbrock *et al.*, 1985). It increases oestradiol concentrations in immature female rats which is associated with increased aromatase activity and is preceded by decreased 5-alpha-reductase activity (Uilenbrock *et al.*,

1985). Therefore, it is possible to postulate that the main effect of hCG on the mammary gland is mediated through stimulation of production of ovarian oestrogens and progesterone which in turn play an important role in tumour growth (Dao *et al.*, 1959, 1960; Grubbs *et al.*, 1983*b*, 1986; Huggins *et al.*, 1961, 1962; McCormick *et al.*, 1965, 1973; Welsch, 1985).

Even though the mechanism of action of hCG remains to be explored, our results indicate that hCG in adequate doses may be a suitable hormone in breast cancer prevention and tumour progression after carcinogenic initiation. This knowledge eliminates the uncertainty of whether hCG treatment stimulates a process that had been already initiated at the time of hormonal treatment.

This study was supported by American Cancer Society Grant Number BC-621 and an Institutional Grant from the United Foundation of Greater Detroit.

### References

ASA, S.L., BAYLEY, T.A., KOVACS, K. & HORVATH, E. (1984). Leydig cell hyperplasia due to testicular embryonal carcinoma producing human chorionic gonadotropin. *Andrologia*, **16**, 146.

CHOU, J.Y., WANG, S.S. & ROBINSON, J.C. (1978). Regulation of the synthesis of human chorionic gonadotropin by 5-bromo-2′-deoxyuridine and dibutyryl cyclic AMP in trophoblastic and nontrophoblastic tumor cells. *J. Clin. Endocrinol. Metab.*, **47**, 46.

COCHRAN, B.H., ZULIO, J., BERMA, I.M. & STILES, C.D. (1984). Expression of the c-fos gene and a fos-related gene stimulated by platelet-derived growth factor. *Science*, **226**, 1080.

CONTRACTOR, S.F. & DAVIES, H. (1973). Effect of human chorionic somato mammotrophin and human chorionic gonadotrophin on phytohaemagglutinin-induced lymphocyte transformation. *Nature (New Biol.)*, **243**, 284.

CZERWIEC, F.S., MEIMER, M.H. & PUITT, D. (1989). Transiently elevated levels of c-fos and c-myc oncogene messenger ribonucleic acids in cultured murine Leydig tumor cells after addition of human chorionic gonadotropin. *Mol. Endocrinol.*, **3**, 105.

DAO, T.L., BOCK, F.G. & GREINER, M.J. (1960). Mammary carcinogenesis by 3-methylcholanthrene II. Inhibitory effect of pregnancy and lactation on tumor induction. *J. Natl Cancer Inst.*, **25**, 991.

DAO, T.L. & SUNDERLAND, H. (1959). Mammary carcinogenesis by 3-methylchol anthrene I. Hormonal aspects in tumor induction and growth. *J. Natl Cancer Inst.*, **23**, 567.

GRUBBS, C.J., HILL, D.L., MCDONOUGH, K.C. & PECKHAM, J.C. (1983*a*). N-Nitroso-N-methylurea-induced mammary carcinogenesis: effect of pregnancy on preneoplastic cells. *J. Natl Cancer Inst.*, **71**, 625.

GRUBBS, C.J., JULIANA, M.M., HILL, D.L. & WHITAKER, L.M. (1986). Suppression by pregnancy of chemically induced preneoplastic cells of the rat mammary gland. *Anticancer Res.*, **6**, 1395.

GRUBBS, C.J., PECKHAM, J.C. & MCDONOUGH, K.D. (1983*b*). Effect of ovarian hormones on the induction of 1-methyl-1-nitrosourea-induced mammary cancer. *Carcinogenesis*, **4**, 495.

GULLINO, P.M., PETTIGREW, H.M. & GRANTHAM, F.H. (1975). N-nitrosomethylurea as mammary gland carcinogen in the rat. *J. Natl Cancer Inst.*, **54**, 401.

HUGGINS, C., GRAND, L.C. & BRILLANTES, F.P. (1961). Mammary cancer induced by a single feeding of polynuclear hydrocarbons and its suppression. *Nature*, **189**, 204.

HUGGINS, C., MOON, R. & MORII, S. (1962). Extinction of experimental mammary cancer. I. Estradiol-17B and progesterone. *Proc. Natl Acad. Sci. USA*, **48**, 379.

KIMURA, J., KATOH, M., TAYA, K. & SASAMOTO, S. (1983). An inverse relationship between inhibin and follicle stimulating hormone during the periods of ovulation by human chorionic gonadotropin in dioestrous rats. *J. Endocrinol.*, **97**, 313.

MCCORMICK, G.M. & MOON, R.C. (1973). Effect of increasing doses of estrogen and progesterone on mammary carcinogenesis in the rat. *Eur. J. Cancer*, **9**, 483.

MCCORMICK, G.M. & MOON, R.C. (1965). Effect of pregnancy and lactation on growth of mammary tumours induced by 7,12-dimethylbenz(a)anthracene (DMBA). *Br. J. Cancer*, **19**, 160.

MELMED, S. & BRAUNSTEIN, G.D. (1983). Human chorionic gonadotropin stimulates proliferation of Nb2 rat lymphoma cells. *J. Clin. Endocrinol. Metab.*, **56**, 1068.

MOORE, B.P., HAYDEN, T.J. & FORSYTH, I.A. (1981). Mammary-tumour incidence in Sprague-Dawley rats treated with 7,12-dimethylbenz(a)anthracene: effect of pregnancy and lack of effect of unilateral lactation. *Br. J. Cancer*, **44**, 451.

MOVIGLIA, G.A. & RUSSO, J. (1984). Antimitogenic effect of human chorionic gonadotropin (hCG) on rat mammary epithelial cells in culture. *J. Cell Biol.*, **99**, 1439a.

NICHOLSON, R.I., GOTTING, K.E., GEE, J. & WALKER, K.J. (1988). Actions of oestrogens and antioestrogens on rat mammary gland development: Relevance to breast cancer prevention. *J. Steroid Biochem.*, **30**, 95.

RUSSO, I.H. & RUSSO, J. (1986). From pathogenesis to hormone prevention of mammary carcinogenesis. *Cancer Surveys*, **5**, 649.

RUSSO, I.H. & RUSSO, J. (1988). Hormone prevention of mammary carcinogenesis: a new approach in anticancer research. *Anticancer Res.*, **8**, 6.

RUSSO, I.H., TEWARI, M. & RUSSO, J. (1989*a*). Morphology and development of mammary glands rat, including methods of study, collection and preparation of material. In: *Integument and Mammary Gland, Monograph Series on the Pathology of Laboratory Animals*, Jones, T.C., Mohr, U. & Hunt, R.D. (eds) p. 233. Springer-Verlag: Berlin.

RUSSO, J. (1983). Basis of cellular autonomy in the susceptibility to carcinogenesis. *Toxicol. Pathol.*, **11**, 149.

RUSSO, J., GUSTERSON, B., ROGERS, A. & 3 others (1990). Comparative study of human and rat mammary tumorigenesis. *Lab. Invest.*, **62**, 244.

RUSSO, J. & REINA, D. (1985). Human chorionic gonadotropin hormone (hCG) as an inhibitor growth factor on MCF-7 cells. *Proc. Am. Assoc. Cancer Res.*, **26**, 108a.

RUSSO, J. & RUSSO, I.H. (1978). DNA-labeling index and structure of the rat mammary gland as determinants of its susceptibility to carcinogenesis. *J. Natl Cancer Inst.*, **61**, 1451.

RUSSO, J. & RUSSO, I.H. (1980). Influence of differentiation and cell kinetics on the susceptibility of the rat mammary gland to carcinogenesis. *Cancer Res.*, **40**, 2677.

RUSSO, J. & RUSSO, I.H. (1987*a*). Biological and molecular bases of mammary carcinogenesis. *Lab. Invest.*, **57**, 112.

RUSSO, J. & RUSSO, I.H. (1987*b*). Development of human mammary gland. In *The Mammary Gland*, Neville, M.C. & Daniel, C.W. (eds) p. 67. Plenum Press: New York.

RUSSO, J., RUSSO, I.H., ROGERS, A.E. & 2 others (1989*b*). Tumors of the mammary gland. In *Pathology of Tumors of Laboratory Animals, WHO, Vol. 1*, Turusov, V. & Mohr, U. (eds).

RUSSO, J., RUSSO, I.H., VAN ZWIETEN, M.J. & 2 others (1989*c*). Classification of neoplastic and non-neoplastic lesions of the rat mammary gland. In *Integument and Mammary Gland. Monograph Series on the Pathology of Laboratory Animals*, Jones, T.C., Mohr, U. & Hunt, R.D. (eds) p. 275. Springer-Verlag: Berlin.

RUSSO, J., SABY, J., ISENBERG, W.M. & RUSSO, I.H. (1977). Pathogenesis of mammary carcinoma induced in rats by 7,12-dimethylbenz(a)anthracene. *J. Natl Cancer Inst.*, **59**, 435.

RUSSO, J., TAY, L.K. & RUSSO, I.H. (1982). Differentiation of the mammary gland and susceptibility to carcinogenesis. *Breast Cancer Res. Treat.*, **2**, 5.

RUSSO, J., WILGUS, G. & RUSSO, I.H. (1979). Susceptibility of the mammary gland to carcinogenesis. I. Differentiation of the mammary gland as determinant of tumor incidence and type of lesion. *Am. J. Pathol.*, **96**, 721.

SEGAL, S.J. (1980). *Chorionic Gonadotropin*. Plenum Press: New York.

TAY, L.K. & RUSSO, J. (1985). Effect of human chorionic gonadotropin in 7,12-dimethylbenz(a)anthracene-induced DNA binding and repair synthesis by rat mammary epithelial cells. *Chem. Biol. Interact.*, **55**, 13.

UILENBROCK, J.TH.J., MEIJS-ROELOFS, H.M.A., WOUTERSENE, P.J.A. & 4 others (1985). Changes in ovarian steroidogenesis in prepubertal rats induced to ovulate by low doses of human chorionic gonadotropin. *J. Endocrinol.*, **107**, 113.

WELSCH, C.W. (1985). Host factors affecting the growth of carcinogen induced rat mammary carcinomas: a review and tribute to Charles Brenton Huggins. *Cancer Res.*, **45**, 3415.

WIDE, L., HOBSON, B. & WIDE, M. (1980). Chorionic gonadotropin in rodents. In *Chorionic Gonadotropin*, Segal, S.J. (ed.), p. 37. Plenum Press: New York.

ZAR, J.H. (1984). *Biostatistical Analysis*, 2nd ed. Prentice Hall: Englewood Cliffs, NJ.